# Mono- and multi-nucleated ventricular cardiomyocytes constitute a transcriptionally homogenous cell population

**DOI:** 10.1007/s00395-019-0744-z

**Published:** 2019-08-09

**Authors:** Michail Yekelchyk, Stefan Guenther, Jens Preussner, Thomas Braun

**Affiliations:** 1 Department of Cardiac Development and Remodeling, Max Planck Institute for Heart and Lung Research, Bad Nauheim, Germany; 2 German Centre for Cardiovascular Research (DZHK), Partner Site Rhein-Main, Frankfurt am Main, Germany

**Keywords:** Single-cell RNA-sequencing, Multi-nucleated cardiomyocytes, Cardiac hypertrophy, Hypoxic responses, HIF1α

## Abstract

Individual adult ventricular cardiomyocytes are either mono- or multi-nucleated and undergo morphological changes during cardiac hypertrophy. However, corresponding transcriptional signatures, reflecting potentially different functions or the ability for cell-cycle entry, are not known. The aim of this study was to determine the transcriptional profile of mono- and multi-nucleated adult cardiomyocytes by single-cell RNA-sequencing (scRNA-seq) and to investigate heterogeneity among cardiomyocytes under baseline conditions and in pressure-induced cardiac hypertrophy. We developed an array-based approach for scRNA-seq of rod-shaped multi-nucleated cardiomyocytes from both healthy and hypertrophic hearts. Single-cell transcriptomes of mono- or multi-nucleated cardiomyocytes were highly similar, although a certain degree of variation was noted across both populations. Non-image-based quality control allowing inclusion of damaged cardiomyocytes generated artificial cell clusters demonstrating the need for strict exclusion criteria. In contrast, cardiomyocytes isolated from hypertrophic heart after transverse aortic constriction showed heterogeneous transcriptional signatures, characteristic for hypoxia-induced responses. Immunofluorescence analysis revealed an inverse correlation between HIF1α^+^ cells and CD31-stained vessels, suggesting that imbalanced vascular growth in the hypertrophied heart induces cellular heterogeneity. Our study demonstrates that individual mono- and multi-nucleated cardiomyocytes express nearly identical sets of genes. Homogeneity among cardiomyocytes was lost after induction of hypertrophy due to differential HIF1α-dependent responses most likely caused by none-homogenous vessel growth.

**Electronic supplementary material:**

The online version of this article (10.1007/s00395-019-0744-z) contains supplementary material, which is available to authorized users.

## Introduction

Cardiomyocytes are the workhorses of the heart, contracting continuously approximately 3 billion times over an average human lifetime without tiring [23]. Maturation of cardiomyocytes during postnatal development is associated with increased ploidy, i.e., an increase in DNA content. In the adult mouse heart, approximately 85% of all ventricular cardiomyocytes contain two nuclei (2 × 2c) [27]. So far, the reasons for increased polyploidy of cardiomyocytes are unknown, although numerous hypotheses have been proposed including prevention of unwanted cell proliferation [16, 17]. In fact, experimental polyploidization of zebrafish cardiomyocytes is sufficient to inhibit proliferative responses during heart regeneration [8].

Technical limitations have prevented systematic studies about transcriptional heterogeneity among cardiomyocytes, which would provide insights into potential differences between diploid and polyploid cardiomyocytes. Although single-cell RNA-sequencing (scRNA-seq) has become enormously popular during recent years [6], intact mono- and multi-nucleated cardiomyocytes have been mostly exempted, primarily because of their large size up to a length of 200 µm and difficulties to separate cells based on ploidy [23]. Common microfluidic or droplet-based scRNA-seq methods preclude analysis of very large cells [11, 31]. To circumvent the size-problem, isolated cardiomyocyte nuclei have been sequenced [21], but such approaches exclude actively translated mRNAs defining the biological activity of cardiomyocytes. In addition, information about numbers of nuclei within cardiomyocytes and cell quality is lost in any droplet-based scRNA-seq methods unless cardiomyocytes are pre-sorted into different classes of cells.

Here, we describe a novel method, based on the ICELL8 single-cell system, for single-cell RNA-sequencing of intact mono- and multi-nucleated, rod-shaped cardiomyocytes isolated from normal and hypertrophic hearts.

## Results

### Image-based tracking of individual cardiomyocytes for scRNA-seq prevents artificial cell clustering

**Fig. 1:**
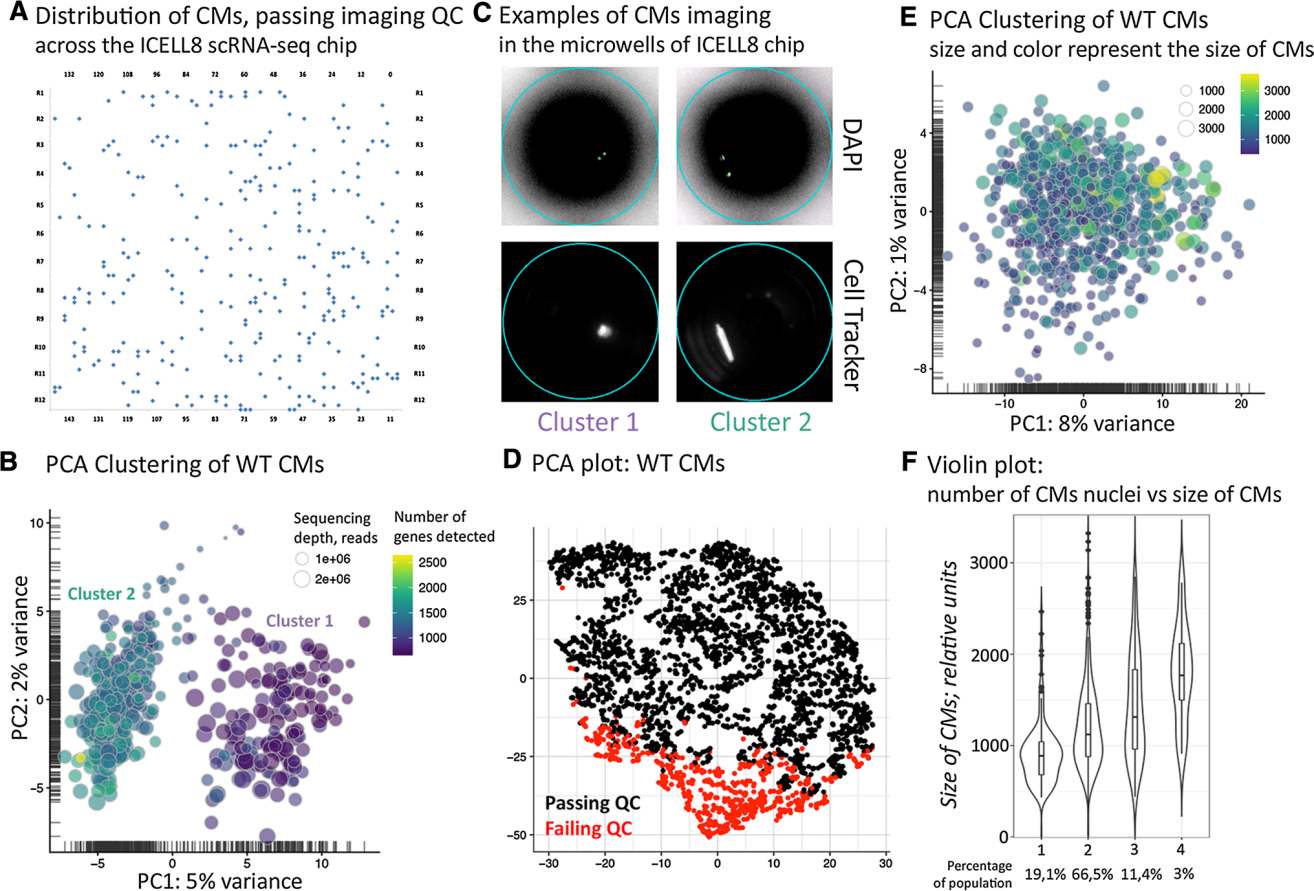
Image-based tracking of individual cardiomyocytes for scRNA-seq prevents artificial cell clustering. **a** Distribution of cardiomyocytes passing imaging QC across the ICELL8 scRNA-seq micro-well chip. **b** Initial PCA clustering of unsupervised data shows clustering of cardiomyocytes into two subgroups. **c** Images of cardiomyocytes in cluster 1 and 2 before library preparation [two-channel reflection mode: DAPI and Cell Tracker (Texas Red)]. Quality control reveals that cells in cluster 1 are damaged. **d** PCA plot showing cardiomyocyte clustering based on QC parameters. **e** PCA plot of intact rod-shaped cardiomyocytes. **f** Violin plot indicating correlation between size and nuclearity of cardiomyocytes

Since common microfluidic or droplet-based scRNA-seq methods do not allow scRNA-seq of whole cardiomyocytes, we employed the ICELL8 platform, which uses a large-bore nozzle dispenser to distribute single cells into 5184 nanowells for further processing. Due to the high physical density of cardiomyocytes, several modifications of the standard protocol were necessary to accomplish adequate loading of nanowells according to the Poisson distribution (Suppl. Fig. 1A, B). Critical steps included frequent dispensing with intermittent gentle mixing of the cell suspension to avoid sedimentation of cardiomyocytes and a reduction of the calculated cell concentration from 1 to 0.2 cell per dispense volume (= 50 nl). Usually, we achieved a loading of 450–750 intact cardiomyocytes per chip, which is below the theoretical value of ~ 1800 cells but still within an acceptable range (Fig. 1a). We assume that high fragility of adult cardiomyocytes and low concentrations of cells are main reasons for suboptimal loading.

Initial analysis of scRNA-seq data from 586 cardiomyocytes suggested the existence of two distinct classes of cardiomyocytes, which clustered separately in PCA and t-SNE plots (Fig. 1b, Suppl. Fig. 1C). However, we noted that clustering was mostly driven by strong differences in the absolute number of genes detected per cardiomyocyte (Fig. 1b). Since the ICELL8 approach includes an imaging step ensuring processing only of nanowells containing single cells, we correlated images of individual cardiomyocytes with the outcome of sequencing reactions and localization in clusters 1 and 2. All cells within cluster 1, characterized by low numbers of detected genes, had a small size, were not rod-shaped anymore, and showed undefined cellular silhouettes, which results from leakage of the Cell Tracker dye (Fig. 1c). We concluded that such cardiomyocytes had encountered cellular damage causing partial RNA degradation, which eventually leads to inefficient RNA-seq library preparation and decreased numbers of detected transcripts. Thus, we changed the experimental design and excluded all cells from subsequent analysis showing any signs of cellular damage.

In a second set of experiments, only scRNA-seq data from undamaged individual cardiomyocytes were analyzed as indicated by image-based analysis. Additional quality control criteria included the total number of detected genes (“features”), percentage of dropout, percentage of mitochondrial transcripts, percentage of non-unique alignments, and presence of cardiomyocyte markers (Suppl. Fig. 1D). In total, we analyzed 2767 micro-wells containing cardiomyocytes, of which 715 harbored a single intact rod-shaped cardiomyocyte (Fig. 1d). After cell lysis, reverse transcription, barcoding, cDNA amplification, library preparation, and sequencing, we obtained 0.6 M reads and the detection of 3.9 k genes per cell on average.

Bioinformatical evaluation using the “scater” R package [12] and PCA analysis revealed only a low degree of heterogeneity (8% and 1% variance for first and second principle components, respectively) among adult cardiomyocytes of healthy mice, which was not sufficient to drive formation of distinct clusters (Fig. 1e, Suppl. Fig. 1E). Remarkably, different cell sizes had no effects on the distribution of cardiomyocytes within the PCA cluster (Fig. 1e, Suppl. Fig. 1E). Taken together, our findings suggest that cardiomyocytes of different sizes are remarkably homogenous and do not form distinct subpopulations. Furthermore, our data indicate that rigorous quality control, which, in case of highly fragile cardiomyocytes, needs to comprise image-based assessment, is essential to avoid technical artifacts that might suggest non-existing heterogeneity.

### Mono- and multi-nucleated cardiomyocytes express similar sets of genes

**Fig. 2:**
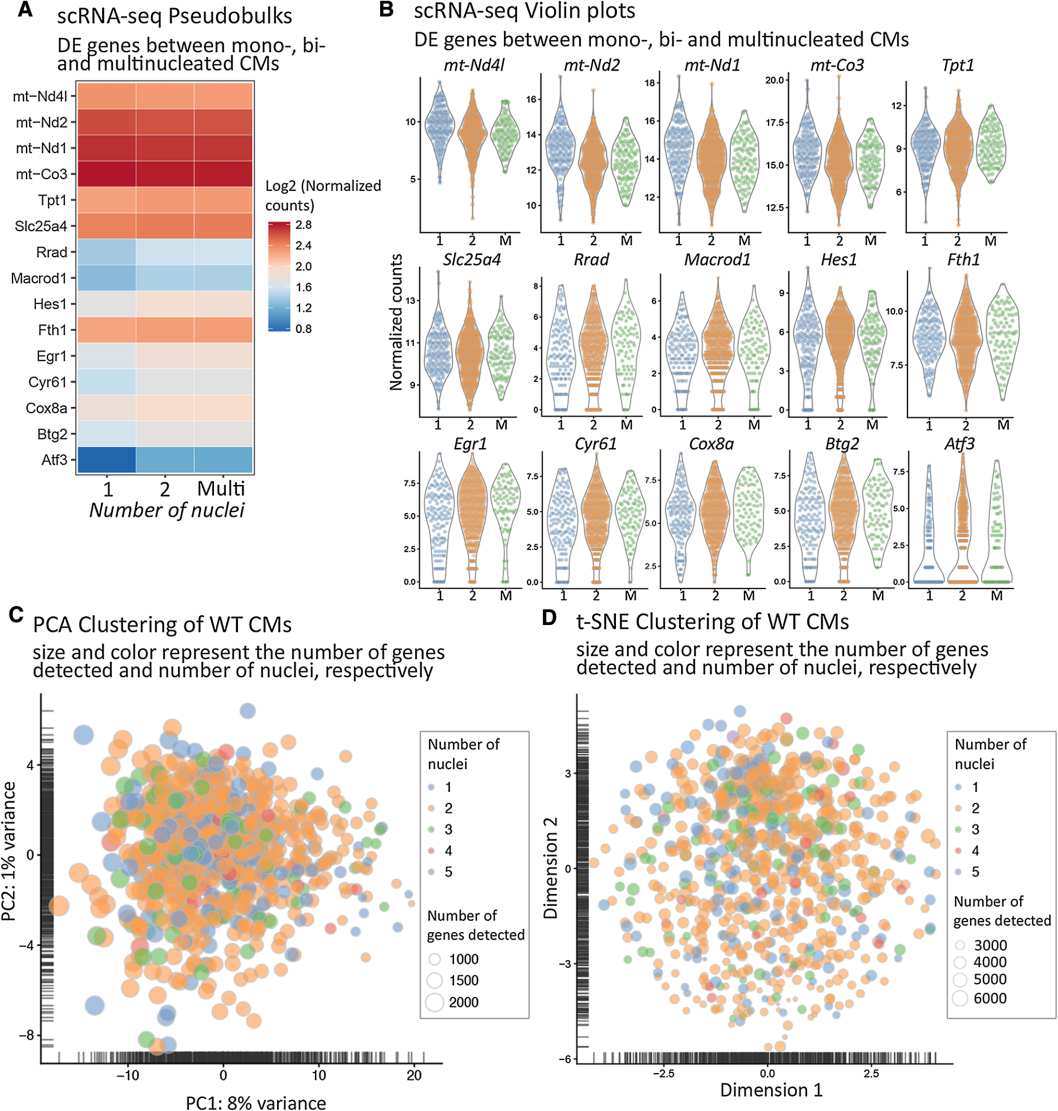
Mono- and multi-nucleated cardiomyocytes express similar sets of genes. **a** Pseudobulk heatmap of top differentially expressed genes in mono-/bi- and mono-/multi-nucleated cardiomyocytes (FDR < 5%). **b** Single-cell violin plots of top differentially expressed genes in mono-/bi- and mono-/multi-nucleated cardiomyocytes (FDR < 5%). “Normalized counts” refer to sequence counts after size-factor normalization. **c**, **d** Numbers of cardiomyocytes nuclei do not drive cell clustering in PCA and t-SNE plots

Multi-nucleated cardiomyocytes are assumed to be larger than mono-nucleated cardiomyocytes [1]. To corroborate these reports and validate our own data, we plotted the size of cardiomyocytes in relation to the number of nuclei. As expected, we observed a clear correlation of size and nuclei numbers (Fig. 1f, Suppl. Fig. 1F). The lack of substantial transcriptional heterogeneity among differentially sized cardiomyocytes already indicated that the number of nuclei has only marginal effects on the transcriptome of individual cardiomyocytes. To explore potential differences or similarities between mono- and multi-nucleated cardiomyocytes in more detail, we separated cardiomyocytes into groups based on the number of nuclei taking advantage of metadata acquired during the procedure. We employed the MAST R package, which detects differentially expressed genes using the Hurdle model for calculation of statistical significances at the single-cell level [5]. In addition to single-cell violin plots, we applied pseudobulk visualization to depict results analogous to more familiar, conventional bulk RNA-seq (Fig. 2a, b). The number of nuclei did not correlate with the number of mapped reads in each group, indicating that similar sequencing qualities and depths were reached thereby excluding a bias due to the RNA content of cardiomyocytes (Suppl. Fig. 1G, H).

**Fig. 3:**
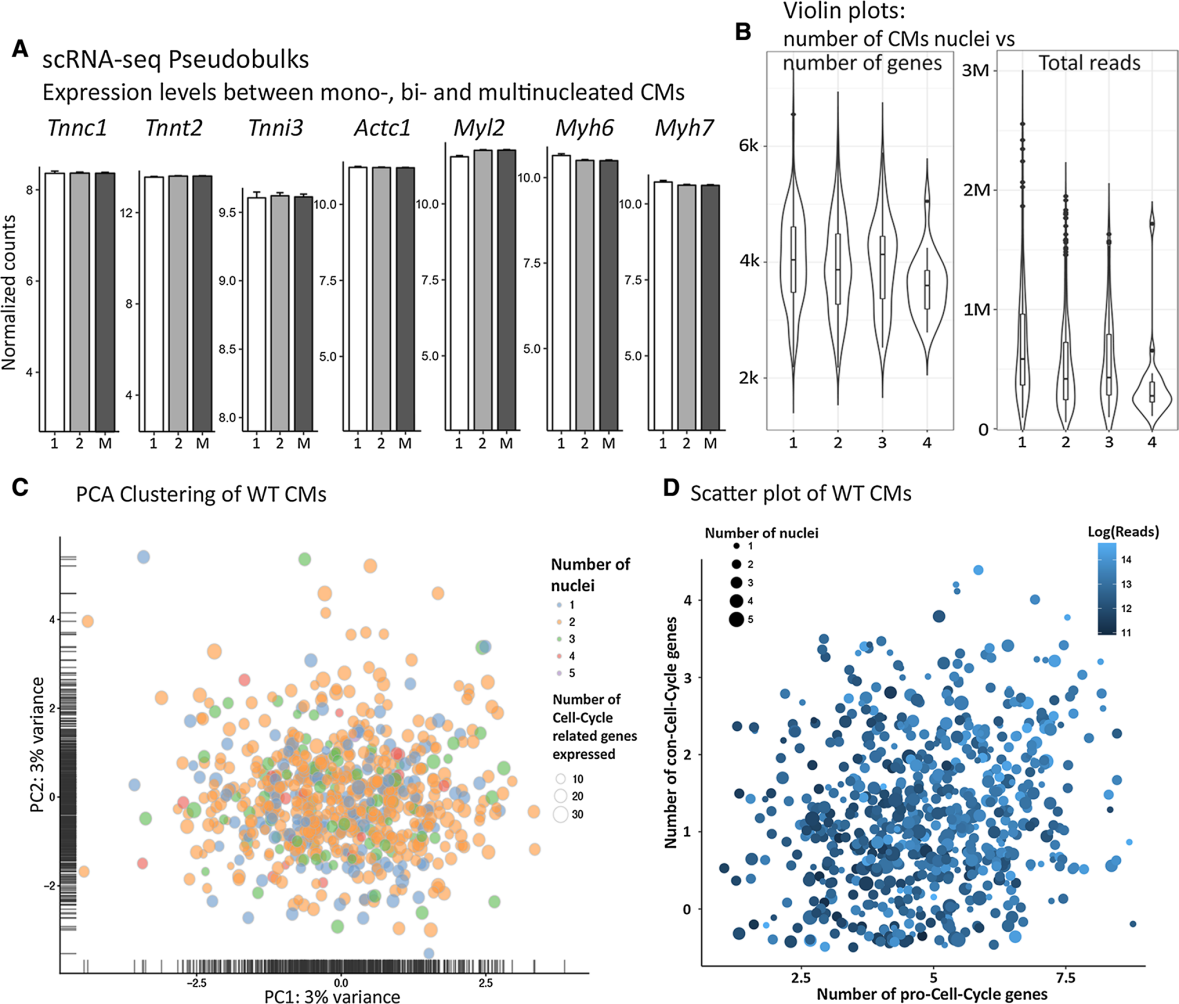
Mono- and multi-nucleated cardiomyocytes stochastically express a restricted set of cell-cycle genes. **a** Pseudobulk barplots demonstrate similar expression levels of cardiac genes in mono- and multi-nuclear cardiomyocytes. “Normalized counts” refer to sequence counts after size-factor normalization. **b** Violin plots exclude correlations between nuclearity and number of genes detected or nuclearity and total read count per cells. **c** Absence of cell clustering based on number of nuclei (color) and number of expressed cell cycle-related genes (presence of *cyclins*, *cdk*’s, Inhibitors plotted as size of data points) in PCA plots. **d** Scatter plot shows no cell clustering based on expression of cell-cycle genes (*cyclins*, *cdk*’s) and cell-cycle inhibiting genes (inhibitors of *cyclins* and *cdk*’s), number of total reads (color), and number of nuclei (size). For better visualization, the number of genes was kept discrete, but data points were shifted on both axes with a random number in the range of (− 0.5, 0.5)

Differential gene expression analysis of mono-, bi-, and multi-nucleated cardiomyocytes revealed only a relatively low number of significantly regulated genes (Fig. 2a, b) including Hes1 and Egr1, which have distinct roles in hypoxia responses [28, 33]. Both genes were slightly down-regulated in mono-nucleated cardiomyocytes. However, log-fold-changes of differentially expressed genes between the groups were minor, and the PCA and t-SNE analysis did not identify clustering of cardiomyocytes with different number of nuclei (Figs. 2c, d, 3a). No meaningful and statistically significant enrichments of Gene Ontology terms or pathway were identified, indicating that the number of nuclei has no profound effect on the composition of the cardiomyocyte transcriptome. Surprisingly, the total read count per cell, which corresponds to the initial mRNA content [18], did not differ between mono- and binucleated cardiomyocytes (Fig. 3b), suggesting that the presence of additional nuclei in cardiomyocytes does not lead to a proportional increase of transcripts.

Although rod-shaped cardiomyocytes did not show sufficient transcriptional heterogeneity to generate distinct clusters in PCA or t-SNE plots, we wanted to know whether individual groups of genes were differentially expressed, considering differences in read count numbers per cell higher than 70% of the mean expression across the whole data set as significant. We detected a significant expression of cell-cycle regulating genes such as *cyclins* and *cdk*’s in some cardiomyocytes (Fig. 3c), which is surprising since adult cardiomyocytes barely cycle [32]. The list of detectable (more than five mapped sequencing reads) cell-cycle-related genes included *Ccni, Ccnl2, Ccnk, Ccnd3, Ccnh, Ccny, Ccnd2, Ccnl1, Ccna2, Cdk4,* and *Ccng1* as stimulatory cell cycle; and *Cdkn2d, Cdk2ap1, Inca1,* and *Cdkn1b* as inhibitory cell cycle-related genes. Interestingly, expression of individual cell-cycle regulatory genes was randomly distributed within the population and no individual cardiomyocyte expressed a full set of cell-cycle genes. Moreover, no correlation to the number of nuclei was evident (Fig. 3d).

### Cardiac hypertrophy induces heterogeneous transcriptional responses in cardiomyocytes

**Fig. 4:**
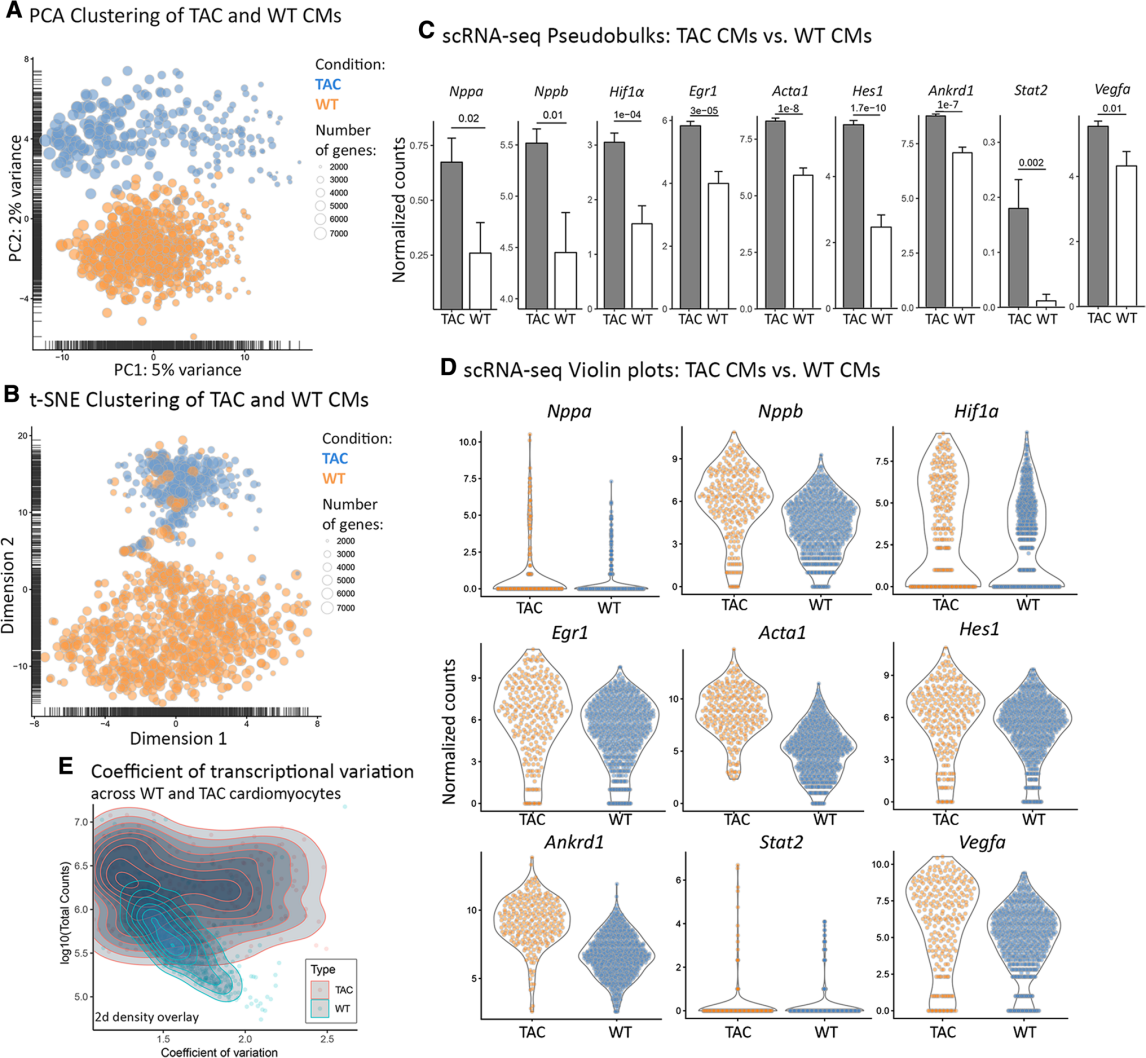
Homogeneity of cardiomyocytes is lost after induction of hypertrophy. **a**, **b** PCA-based and t-SNE-based cell clustering demonstrating transcriptional differences between normal and hypertrophic cardiomyocytes. TAC: *N* = 10 mice; Sham: *n* = 4 mice. **c**, **d** Pseudobulk barplots and single-cell violin plots showing differential expression of various hypoxia-related genes in normal and hypertrophic cardiomyocytes. “Normalized counts” refer to sequence counts after size-factor normalization. **e** The scatter plot shows increase of transcriptional variation upon TAC-induced cardiac hypertrophy

To investigate whether pathological conditions might induce heterogeneity in rod-shaped cardiomyocytes, we induced cardiac hypertrophy by applying transverse aortic constriction (TAC) [3]. Cardiomyocytes isolated from hypertrophic hearts 8 weeks after TAC were clearly different from cardiomyocytes of healthy hearts (WT) (Suppl. Fig. 2A–D; Fig. 4a, b). Bioinformatical analysis using the MAST package demonstrated expression of cardiac marker genes and revealed that the total number of genes detected per cell was comparable between normal and hypertrophic cardiomyocytes, excluding major technical biases. 184 genes were differentially expressed (FDR < 5%) between WT (basal) and TAC conditions (Suppl. Table 1), which caused a clear separation in the PCA and t-SNE analysis (variances of 5% and 2% in the first and second PCA components, respectively). However, we noted an overlap in the PCA plot, containing cells from both TAC and WT conditions, suggesting that not all cardiomyocytes responded equally to hypertrophy.

**Fig. 5:**
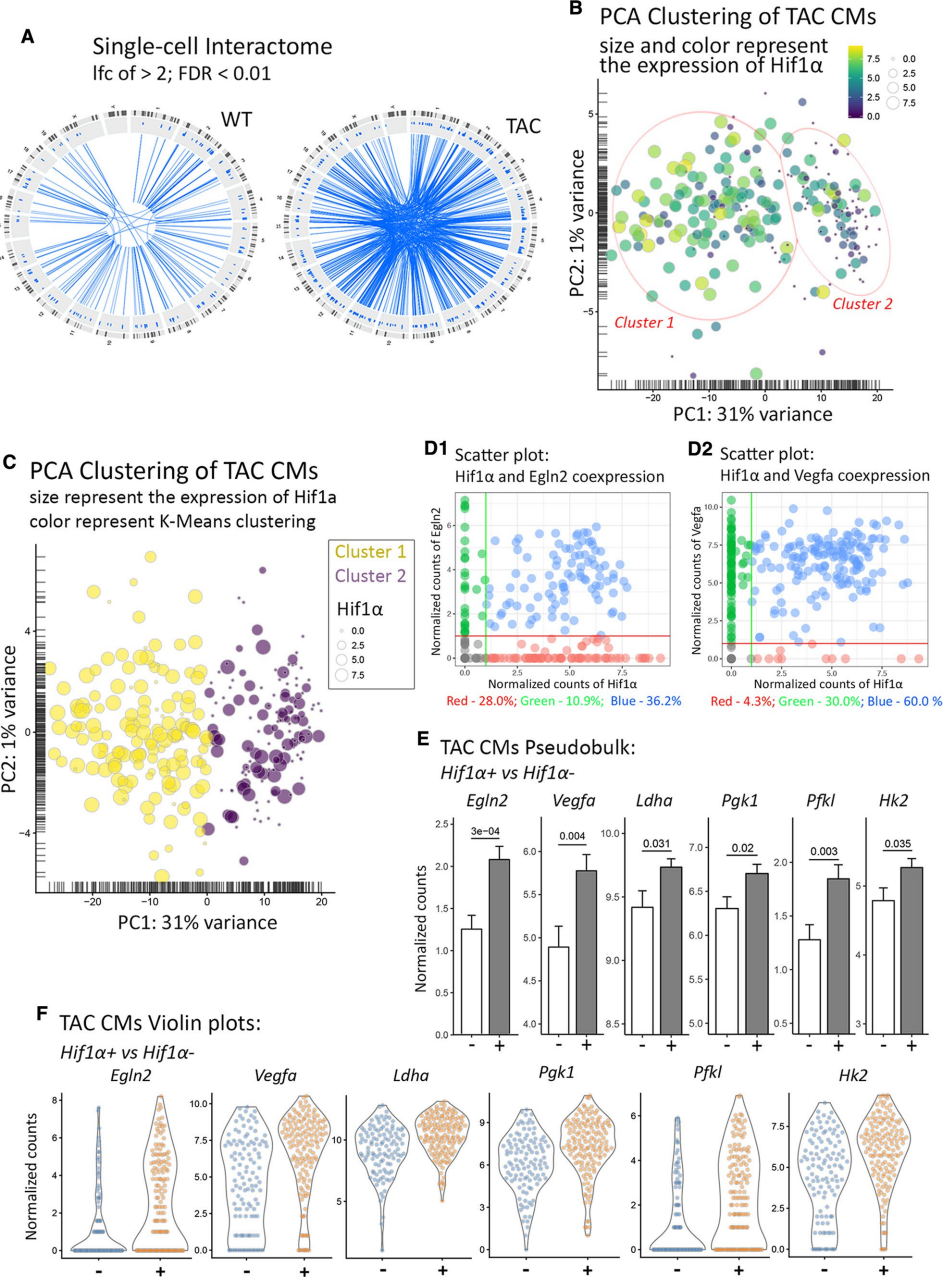
Heterogeneity of cardiomyocytes in hypertrophic hearts is driven by hypoxic responses. **a** Single-cell interactome analysis indicating increase of gene co-expressions during cardiac hypertrophy. Genes showing significant co-expression (excluding base-gene self-pairs) are connected by lines. TAC: *N* = 10 mice; Sham: *n* = 4 mice. **b**, **c** PCA plots of hypertrophic cardiomyocytes identify two cell clusters corresponding Hif1α expression. Red ellipses in **b** represent arbitrary visualizations. The strict definition by *k*-means clustering is given in **c**. **d** Scatter plots indicating co-expression of Hif1α and Egln2 (**d1**) and Hif1α and Vegfa (**d2**) at single-cell resolution. Percentages of cells in different clusters are listed below the plots. **e**, **f** Pseudobulk bar plots and single-cell violin plots demonstrate differential expression of selected genes in Hif1α^+^ and Hif1α^−^ cardiomyocytes (two-tailed *t* test). “Normalized counts” refer to sequence counts after size-factor normalization

Top differentially expressed genes between basal and TAC conditions included hypoxia response and muscle-related genes such as *Nppa*, *Nppb*, *Hif1α*, *Egr1*, *Acta1*, *Hes1*, and *Ankrd1* (Fig. 4c, d). In addition, we found up-regulation of VEGFA and STAT pathway components such as *Stat2*. Likewise, TAC cardiomyocytes were enriched (*p* < 5%) for the GO terms “Hypoxia response via HIF-activation”, as well as for different inflammation- and signaling pathways-related terms (Suppl. Fig. 3A). Furthermore, we calculated cell-associated coefficients of transcriptional variation for both WT and TAC cardiomyocytes, and plotted them to the number of sequencing reads (Fig. 4e). The resulting scatter plot revealed dramatically increased transcriptional variation after TAC-induced cardiac hypertrophy. In addition, we generated single-cell interactome maps (see methods), which also revealed a dramatic increase of gene–gene co-expressions in TAC versus baseline conditions (236 pairs for WT and 716 pairs for TAC) (Fig. 5a; Suppl. Fig. 3B), indicating increased transcriptional activity of cardiomyocytes during hypertrophy.

### Heterogeneity of cardiomyocytes in hypertrophic hearts is driven by hypoxic responses

PCA analysis of hypertrophic cardiomyocytes indicated the existence of two partially connected clusters correlating with *Hif1α* expression (Fig. 5b), which is a crucial transcriptional factor mediating hypoxic responses [22]. Clustering was performed using the *k*-means algorithm (Fig. 5c) [29]. Cardiomyocytes in cluster 1 (“Hif1α^high^”) were transcriptionally more active compared to cluster 2, resulting in a high number of differentially expressed genes between both clusters (Suppl. Table 2). Cluster 1 cardiomyocytes were enriched for “Angiogenesis”, probably as a consequence of *Hif1α* expression, while cluster 2 cardiomyocytes were enriched for “Striated Muscle Contraction” (Suppl. Fig. 3C). To analyze the impact of *Hif1α* expression on the transcriptional profile of hypertrophic cardiomyocytes, we set a threshold of minimal *Hif1α* expression greater than 70% of the mean expression across the data set. According to this definition, ~ 41% of cardiomyocytes expressed *Hif1α* and ~ 59% did not. Both groups exhibited similar expression levels of *Tnni3*, *Tnnt2*, *Myh6,* and *Myh7* (Suppl. Table 3, Suppl. Fig. 4A) but more than 2000 genes were differential expressed with an FDR < 1% (Suppl. Fig. 4B; Suppl. Table 4). The majority of deregulated genes were found in the Hif1α^high^” group, consistent with higher transcriptional activity in these cardiomyocytes. Furthermore, cardiomyocytes in the Hif1α^high^” group showed higher expression of *Egln2* (also called *Phd1*) [30] and *Vegfa* [24]. The concomitant up-regulation of *Egln2* and *Vegfa* in cluster 1 was clearly evident by pseudobulk analysis (Fig. 4c) and single-cell visualization (Fig. 4d, e). In addition, cardiomyocytes in the Hif1α^high^” group were enriched for *Ldha, Pgk1, Pfkl,* and *Hk2* transcripts (Fig. 5f), which are known targets of *Hif1α*. No differences in average numbers of nuclei were found in Hif1α^+^ compared to Hif1α^−^ cardiomyocytes (Suppl. Fig. 4C).

### HIF1α stabilization in cardiomyocytes inversely correlates with distance to vessels in hypertrophic hearts

**Fig. 6:**
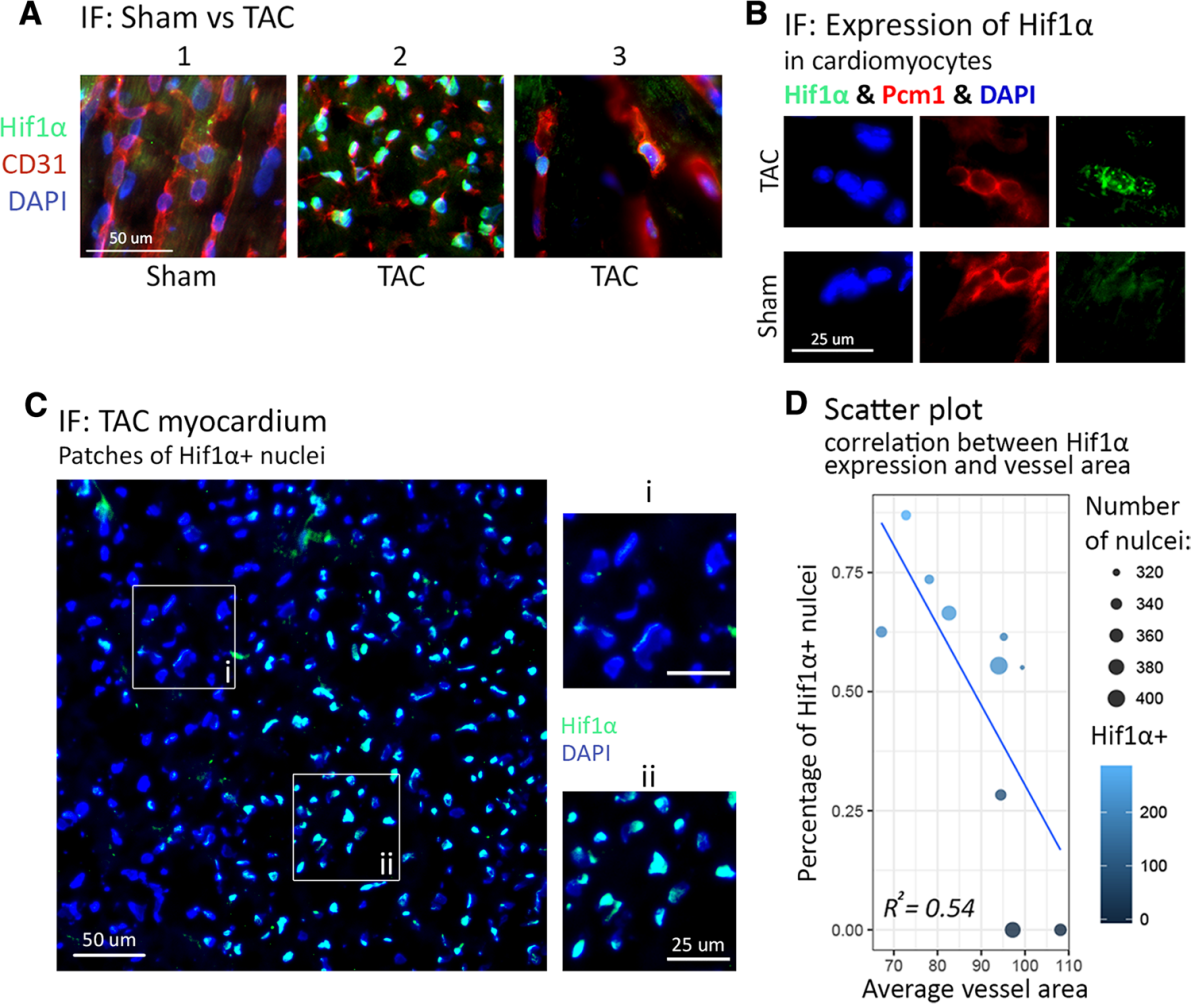
Hif1α stabilization in cardiomyocytes inversely correlates with distance to vessels in hypertrophic hearts. **a** Immunostaining for Hif1α and CD31 under different conditions: (1) Sham; (2, 3) TAC. TAC: *N* = 10 mice; Sham: *n* = 4 mice. **b** Immunostaining for Hif1α and Pcm1. **c** “Patchy” structure of Hif1α expression. **d** Scatter plot showing inverse correlation of Hif1α expression in cardiomyocytes and average vessel area (square µm) in the proximity (*R*^2^ = 0.54)

Since our data indicated that a substantial amount of the cardiomyocyte heterogeneity in hypertrophic hearts might be driven by hypoxic responses, we wondered whether during hypertrophic growth, some areas of the myocardium encounter low oxygen levels probably due to heterogeneous vessel growth. Cells undergoing hypoxic responses were detected by immunofluorescence staining for HIF1α and vascularization was assessed by staining for the endothelial cell marker CD31. The normal heart did not show HIF1α expression in nuclei under baseline conditions and was characterized by a well-organized vascular network with wide and long blood capillaries (Fig. 6a1). In contrast, hypertrophic hearts contained areas with patches of endothelial Hif1α^+^ nuclei located in substantially smaller capillaries lacking obvious interconnections (Fig. 6a2, a3). Co-staining with the nuclear cardiomyocyte marker PCM1 [9] revealed that such areas also contained cardiomyocytes undergoing hypoxic responses as indicated by HIF1α localization in nuclei (Fig. 5b). Expression of HIF1α was inhomogeneous and showed a “patchy” pattern (Fig. 6c). To quantify the inverse correlation between HIF1α expressing cardiomyocytes and the presence of vessels in the proximity, we counted the number of Hif1α^+^ cells as well as the average area of blood vessels (CD31^+^ area) per view field. A correlation of *R*^2^ = 0.54 was calculated between both parameters confirming that HIF1α-expressing cells are preferentially located in areas with a comparatively low degree of capillarization (Fig. 6d).

## Discussion

Our study demonstrates that adult rod-shaped cardiomyocytes are relatively homogenous, and that even mono-, bi-, and multi-nucleated cardiomyocytes express nearly identical sets of genes. In contrast, induction of hypertrophy induced strong transcriptional changes in cardiomyocytes and generated substantial heterogeneity. Since heterogeneity of cardiomyocytes in this pathological condition was mainly driven by hypoxic responses and nuclear HIF1α-expression was inversely correlated with capillarization of the myocardium, we conclude that heterogenous vessel growth is the main reason for cardiomyocyte heterogeneity in the remodeling hypertrophic heart. Our findings indicate that differences in the transcriptional signatures of individual rod-shaped adult cardiomyocytes depend more on the local tissue microenvironment than on ploidy or other endogenous features, although we cannot exclude that, e.g., differences in the chromatin landscape, post-translational modifications, or others contribute to the differential response of individual cardiomyocytes under pathological conditions.

It is known for a long time that most types of pathological hypertrophy result in a lower capillary density, particularly in the subendocardial region, because growth of the capillary bed does not keep pace with increases in cardiac mass, thereby increasing the path length for oxygen transport [25]. During the physiological phase of adaptive cardiac growth angiogenesis is enhanced due to up-regulation of mTOR and expression of HIF1a-responsive angiogenic factors including VEGF, which promote coordinated cardiomyocyte growth and angiogenesis [14, 26]. Furthermore, at early states of hypertrophy, local hypoxia will induce hypoxic responses and expression of *Vegfa* in cardiomyocytes, which will promote local angiogenesis as an attempt to restore normal oxygenation throughout the myocardium. In pathological conditions, HIF1α is down-regulated, probably via up-regulation of p53 and MDM2-dependent ubiquitylation, resulting in proteasomal degradation of HIF1α, which causes a mismatch of cardiomyocyte growth and capillary density [19, 20]. Interestingly, we observed an enrichment of the GO term “Hypoxia response via HIF-activation” in a subset of individual cardiomyocytes by single-cell RNA-seq after TAC, which was also one of the main drivers for the induction of cardiomyocyte heterogeneity. The heterogeneity of cardiomyocytes after TAC in response to hypoxic signaling indicates that degradation of HIF1α, even at advanced stages of pathological remodeling (8 weeks after TAC), does not occur in all cardiomyocytes, but is withheld in a subset of cells, which still show physiological responses. Currently, we do not know whether this feature reflects ongoing continuous disruption of coordinated tissue growth and angiogenesis in the heart or results from different endogenous properties of individual cardiomyocytes, independent of transcriptional similarities. We reason that the newly acquired knowledge about heterogenous responses of cardiomyocytes under pathological conditions might provide new clues to prevent the progression from adaptive cardiac hypertrophy to heart failure [13].

Aside from hypoxic response-driven cardiomyocyte heterogeneity, we found that rod-shaped cardiomyocytes form a very homogenous cell population, which was surprising, since we expected strong effects of the number of nuclei on transcriptional profiles. The absence of transcriptional heterogeneity between cardiomyocytes might suggest that polyploidization is a byproduct of terminal differentiation, prevents unwanted cell proliferation, and/or serves other unknown functions. It had also been claimed that multi-nucleation allows organ growth without deteriorating changes in cell shape and function, results in higher metabolic activity and gene expression, or enables better stress responses [16, 17]. The induction of heterogeneity by hypoxic stress might indicate that polyploidization allows better adaptation to chronic stress and injury [17], although we did not find any differences between mono- and multi-nucleated cardiomyocytes in this respect. Likewise, mono- and multi-nucleated cardiomyocytes showed the same random expression of single-cell cycle-related genes, which does not argue in favor of the idea that mono-nuclear cardiomyocytes own a higher propensity to enter cell cycling. Since we observed a clear correlation between the number of nuclei and cell size, but no increase of the number of reads per cell, we reason that increased ploidy does not necessarily leads to increased gene expression.

Of course, our study has some limitations. (1) We restricted the analysis to rod-shaped cardiomyocytes, thereby excluding cardiomyocytes of unconventional shape, e.g., round-shaped or immature cardiomyocytes [13]. (2) scRNA-seq does not provide a comprehensive assessment of all transcripts in a cell, but is limited to ~ 15 to 30% of the transcriptome. (3) Individual cardiomyocytes might differ in respect to epigenetic landscape, chromatin accessibility, post-translational modifications, or other features that might contribute to regional differences (epi/endocardium; apex/base). (4) The nanowell-based approach limits the number of analyzed cells, although image-based quality control eradicates artifacts perturbing scRNA-seq analysis. Such limitations might have prevented detection of heterogeneity among cardiomyocytes or in cardiomyocytes lacking the canonical rod-shaped morphology. Moreover, it is entirely possible that mono- and multi-nucleated ventricular, rod-shaped cardiomyocytes differ with respect to lowly expressed genes, which are below the threshold of what can currently be detected by scRNA-seq. Nevertheless, our investigation clearly indicates that mono- and multi-nucleated own a remarkably similar transcriptional profile and show similar transcriptional responses to pressure overload. The fabrication of false heterogeneity due to inclusion of damaged cardiomyocytes strongly asks for strict quality control measures to avoid experimental artifacts. We assume that our data set covering intact, rod-shaped mono- and multi-nucleated cardiomyocytes will serve as a valuable resource for future studies.

## Methods

### Mouse experiments

All animal experiments were performed in accordance with German animal protection laws and EU (Directive 2010/63/EU) ethical guidelines and were approved by the local governmental animal protection authority Regierungspräsidium Darmstadt (TVA: B2/1208). Six-month-old C57Bl6 males were used for all experiments. Transverse aortic constriction was accomplished using 26-gauge needles to partially ligate the proximal aorta resulting in an acute left-ventricular pressure overload. Cardiomyocytes were isolated 8 weeks after surgery.

### Single-cell RNA-sequencing of cardiomyocytes

Single-cell suspensions of cardiomyocytes were isolated from the murine heart by enzymatic digestion using a Langendorf system. After perfusion with the enzyme solution, the ventricles (but not the atria) were dissected, cut into smaller pieces and gently triturated [9]. Next, cardiomyocytes were washed 3 × with PBS prior to staining. Cardiomyocytes were counted and stained with blue nuclear and red cytosolic stains (NucBlue Live, Invitrogen, ref. R37610 and CellTracker Red CMTPX, life technologies, USA, ref. C34552). After staining, cell suspension was washed 3 × with PBS (5 min, 200 g). Stained cell suspension contained primarily ventricular cardiomyocytes. Next, cardiomyocytes suspensions were dispensed into barcoded micro-well ICELL8 chips (Takara Bio, USA, cat. 640143). Loading of cardiomyocytes was reduced by 0.2 × compared to the original ICELL8 protocol [7]. Furthermore, three additional pipetting steps of the source suspension were introduced to avoid cell sedimentation. For TAC experiment, 7/8 of wells were loaded with TAC-derived cardiomyocytes and 1/8 of wells were loaded with WT-derived cardiomyocytes to avoid batch effects caused by library preparations. Each well of the chip was photographed in the reflection mode to obtain the images of nuclear and cytosolic staining. Wells containing cells of interest were selected for further library preparation using the ICELL8 Cell-Select software.

### ICELL8 library preparation and sequencing

The RT-mix, including reagents for cell lysis, reverse transcription, first and second strand synthesis (SMARTer technology, Takara Bio, USA, cDNA synthesis kit cat. 634926), and PCR cDNA amplification, was dispensed into selected wells. After incubation and thermal cycling, all amplified cDNAs from selected wells were pooled. Amplified cDNA was used as input material for fragmentation, insertion of Illumina sequencing adapters and additional PCR amplification using the Nextera XT DNA library preparation kit (Illumina, USA, cat. FC-131-1024). Each sub-library contained a unique 10-basepair barcode adapter at one side and Nextera-type N7XX adapters at the other side. After cleanup and SPRI-beads size selection, concentration of DNA in the library was measured by the Qubit-HS assay (Thermo Fischer, USA, cat. Q32854) and the quality checked with LabChip GX Touch 24 (PerkinElmer, USA, cat. CLS138162) using the DNA-3K chip (PerkinElmer, USA, cat. CLS960013). The current protocol is suitable for 3′ end sequencing allowing single-end sequencing. The library was sequenced on the Illumina NextSeq500 high75 cartridge v.2 (cat. FC-404-2005). The length of read was 80 bp from one side (library) and 11 bp from the other side (well barcode).

### Deposition of sequencing data

Raw sequencing data from scRNA-seq were deposited in the European Nucleotide Archive (https://www.ebi.ac.uk/ena) under accession number PRJEB29049.

### Analysis of scRNA-seq data

Using the unique barcode present in each well, reads from each cell were demultiplexed. Mapping was conducted with STAR [4]. An expression matrix was generated (with cells on *X*-axis and genes on *Y*-axis), containing feature (information about genes) and phenotype data (information about cells) in the SCE-Set format [12]. Further analysis was done in the R programming environment. The normalization of counts was performed on pooled counts for multiple cells, effectively reducing the incidence of “problematic” zeros [10]. Raw counts were summed across pools of cells of different sizes, and summed values were used to calculate pool-based size factors. Those size factors were then deconvoluted to infer size factors for individual cells without the need of spike-ins. Therefore, levels of gene expression are referred as “Normalized counts”. PCA and TSNE analysis were used for clustering analysis [2, 15]. MAST package was used for supervised differential expression analysis [5]. Single cells were separated based on the expression of particular genes of interest (e.g., Hif1α), forming groups of gene-positive and gene-negative cells. Clustering was performed using the *k*-means algorithm [29]. Data are shown as mean ± SEM. R and RStudio software were used to determine statistical significance. Two treatment groups were compared by two-tailed Student’s *t* test. Results were considered statistically significant when *p* value (or FDR when applicable) < 0.05. Coefficient of transcriptional variation (CV) was calculated with the following formula: CV_*j*_ = SD (*A*_*j*_)/mean (*A*_*j*_); *i* = [1; *i*_max_], where *A*_*j*_—expression vector from the matrix *A*_*ij*_with *i* rows (transcripts) and *j* columns (cells).

### Construction of single-cell interactome maps

Single-cell interactome data set were generated to allow an unbiased approach for separation of single cells into phenotypically similar groups. We selected genes (“Base-Genes”) that were expressed in less than 80% of cardiomyocytes and showed at least 10% of mean expression levels (2617 genes in total). Base-gene-positive (> 0.7 × mean expression) and negative (< 0.7 × mean expression) groups, containing at least ten cells each, were subjected to differential gene expression analysis using the MAST approach [5] resulting in a list, which relates the 2617 base-genes to differentially expressed genes. Only differentially expressed genes with fold change > 2 and FDR < 0.01 were used for further analysis. Base-genes together with genes showing a statistically significant correlation formed an interactome map visualized in circular plots. Genes showing significant co-expression (excluding base-gene self-pairs) were connected by lines.

### Tissue sectioning and immunostaining

After dissection, hearts were gradually frozen in CryoTek cryopreservation gel and sectioned using a cryostat (12 um slices). Slices were fixed with 4% PFA and blocked (1% BSA (Sigma, Germany), 0.3% Triton X-100 (Sigma, Germany), and 2% FCS (Sigma, Germany) in PBS) for 1 h. Processed slides were incubated with primary antibodies in blocking solution overnight at 4 °C. After three washes with PBS, slides were incubated for 1 h at RT with appropriate secondary antibodies diluted in PBS. After two washes with PBS, slides were stained with DAPI, followed by additional washing with PBS. Coverslips were mounted with Mowiol 4-88 mounting media (Sigma, Germany, cat. 81381). Images were obtaining using a Zeiss Z1 microscope (Zeiss, Germany). Image analysis was conducted with ImageJ software using the “Particle Analyzer” package.

### Antibodies

The following primary antibodies were used in the study: Hif1α—Rabbit polyclonal, Novus Biologicals, USA, NB100-479SS, 1/300 dilution; CD31—Goat polyclonal, Novus Biologicals, USA, AF3628, 1/300 dilution; cTnT—Mouse monoclonal, Abcam, UK, ab8295, 1/300 dilution; Pcm1—Mouse monoclonal, Santa Cruz Biotechnology, USA, sc-398365, 1/300 dilution. The following secondary antibodies were used in the study: Anti-rabbit (donkey) FITC, Sigma, Germany, AP182F, 1/500 dilution; Anti-goat (chicken) Alexa 594, Invitrogen, USA, A21468, 1/500 dilution; Anti-mouse (goat) Alexa 594, Invitrogen, USA, A11005, 1/500 dilution.

## Electronic supplementary material

Below is the link to the electronic supplementary material.

Supplementary Information

Supplementary material 1
