# Supplementary material for: Mono- and multi-nucleated ventricular cardiomyocytes constitute a transcriptionally homogenous cell population
Source: Basic Res Cardiol. 2019 Aug 9;114(5):36. doi: 10.1007/s00395-019-0744-z (PMC6689038; doi:10.1007/s00395-019-0744-z)
Supplement: Supplementary file 1 — Supplementary Information [file 395_2019_744_MOESM1_ESM.pdf]

Supplementary Information

Mono- and multi-nucleated ventricular cardiomyocytes constitute a transcriptionally homogenous cell population

Michail Yekelchyk[1], Stefan Guenther[1], Jens Preussner[1], and Thomas Braun[1,2, 3]

[1] Department of Cardiac Development and Remodeling, Max Planck Institute for Heart and Lung Research, Bad Nauheim, Germany

[2] German Centre for Cardiovascular Research (DZHK), Partner site Rhein-Main, Frankfurt am Main, Germany

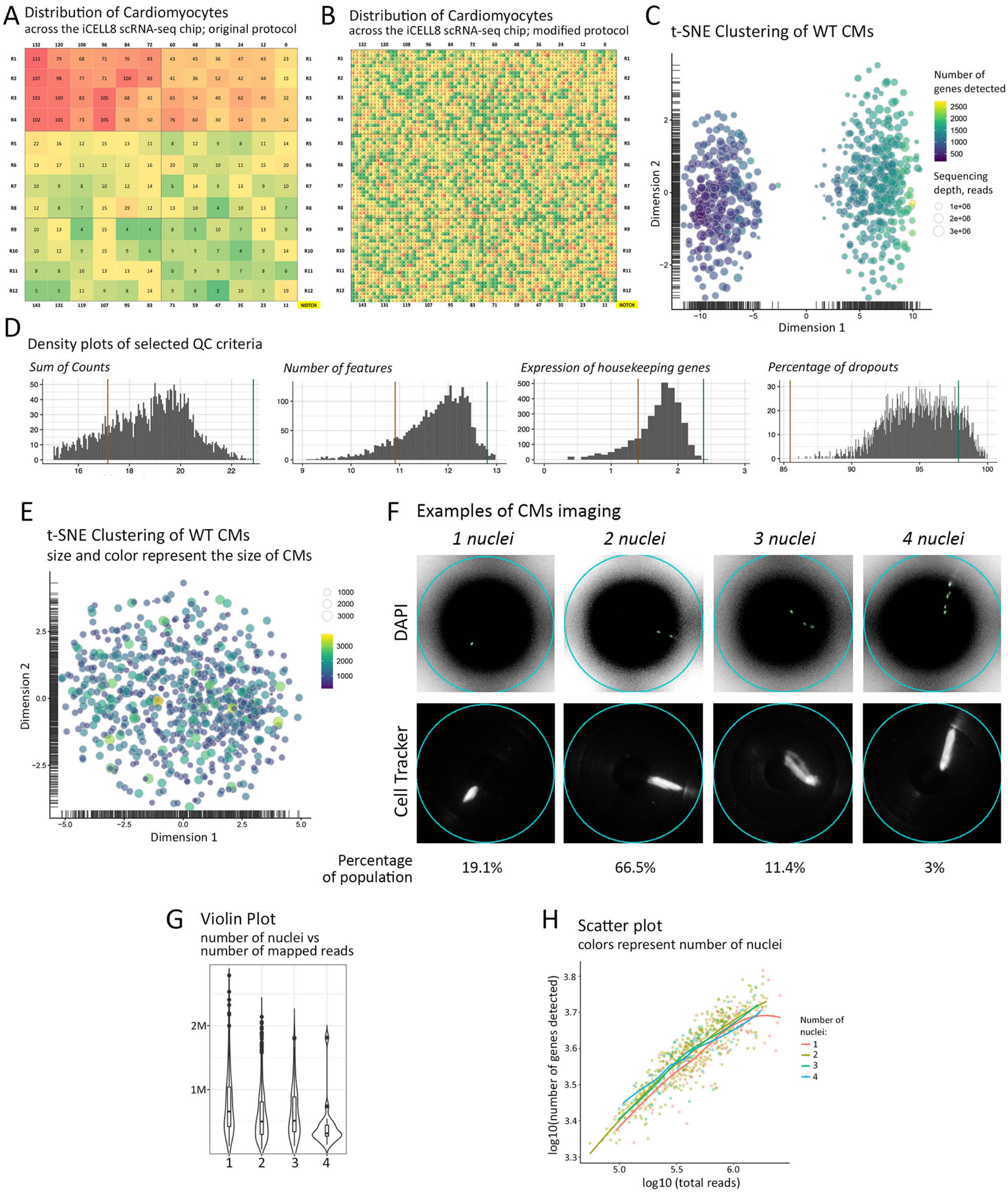

Supplementary Figure 1. Dispensing maps and quality criteria for scRNA-seq. (A) The default dispensing protocol results in heterogenous distribution of cardiomyocytes due to the high sedimentation rate of cardiomyocytes; colors represent the amounts of cells in the clusters of micro-wells. **(B)** The modified dispensing protocol homogeneously distributes cardiomyocytes on the chip; colors represent the amounts of cells in the micro-wells. **(C)** Initial t-SNE clustering of unsupervised data shows clustering of cardiomyocytes into two subgroups. **(D)** Density plots showing the thresholds and cut-offs for selected QC parameters. **(E)** t-SNE plot of intact rod-shaped cardiomyocytes. **(F)** Exemplary images of cardiomyocytes with different numbers of nuclei before library preparation (2 channel reflection mode: DAPI and Cell Tracker (Texas Red). **(G)** Violin plots exclude correlations between nuclearity and number of mapped reads per cell. **(H)** Scatter plot demonstrating linear correlation of the number of genes detected and number of total reads in log space, as well as the random distribution of cardiomyocyte nuclearity in the plot.

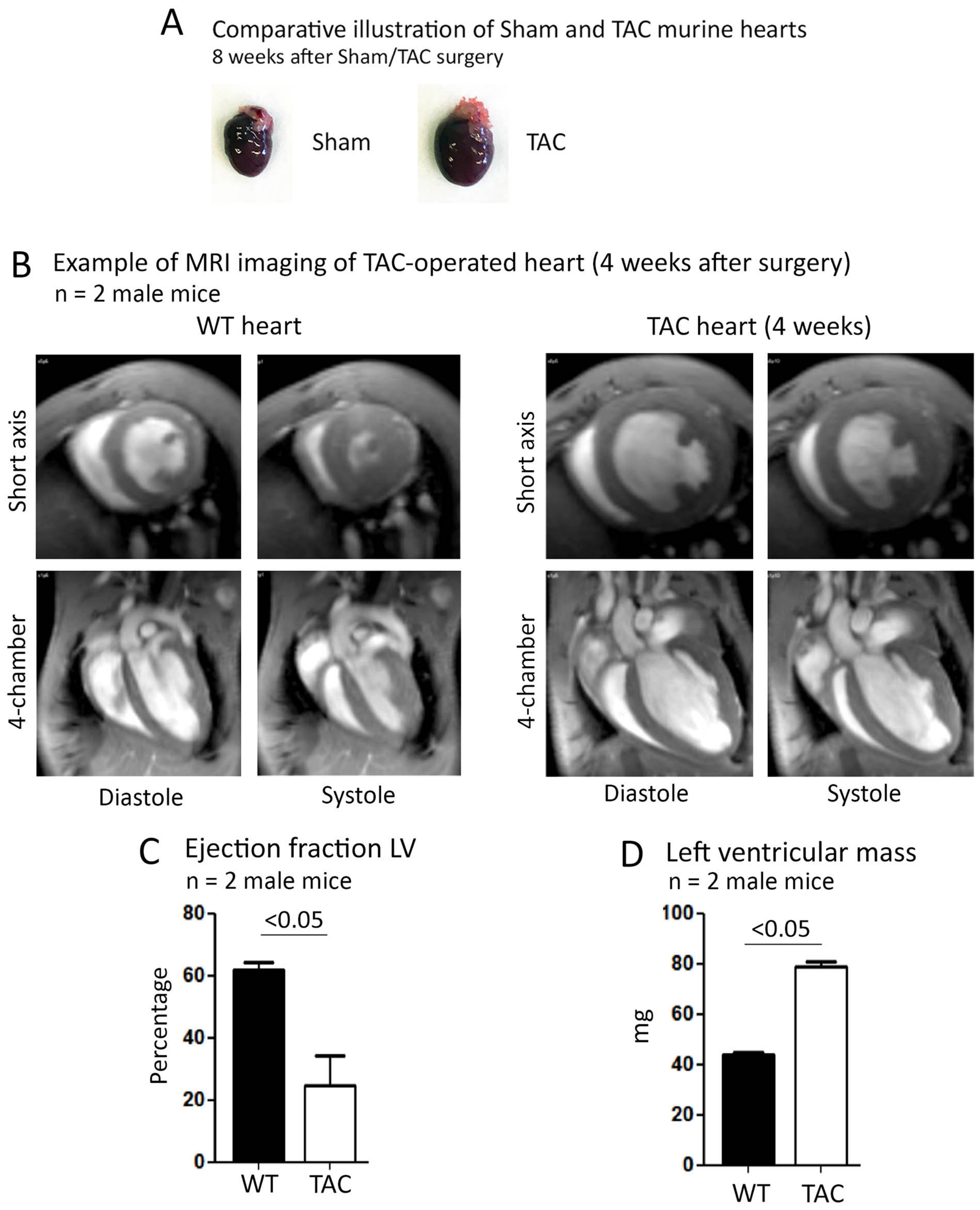

Supplementary Figure 2. Effects of transaortic constriction on cardiac hypertrophy and function. (A) Macroscopic images of isolated murine hearts 8 weeks after transaortic constriction (TA) and sham operation. **(B-D)** Assessment of left ventricular mass and ejection fraction by cardiac magnetic resonance imaging (MRI) of murine hearts 4 weeks after sham operation (WT) and TAC. TAC operation induces profound cardiac hypertrophy (n=4, two-tailed Student's t-test). The images in (B) represent short axis (top) and 4-chamber (bottom) views of sham and TAC-operated hearts in systole and diastole.

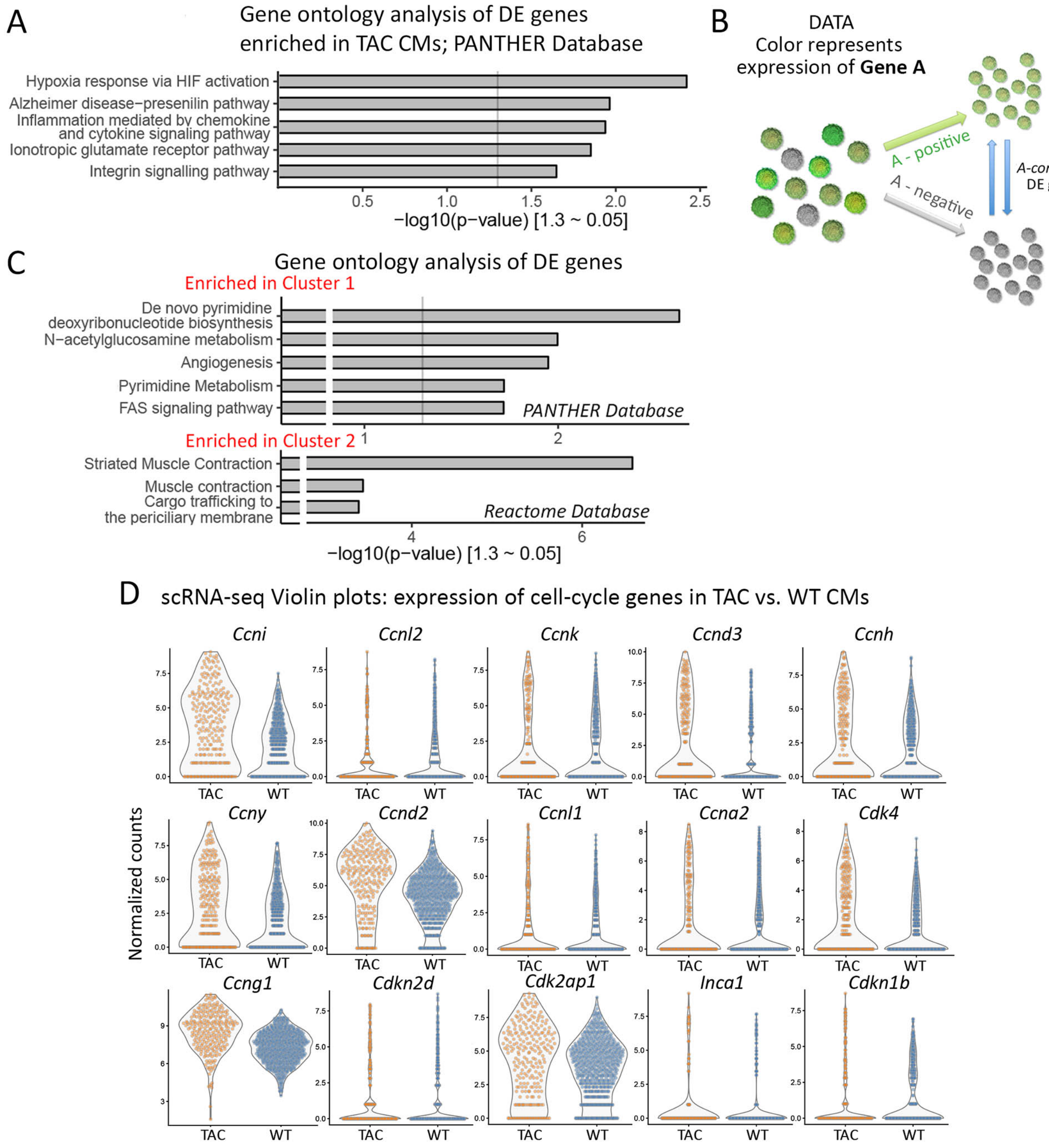

Supplementary Figure 3. Cardiac hypertrophy induces heterogeneous transcriptional responses in cardiomyocytes. (A) Enriched Gene Ontology terms of top differentially expressed genes in cardiomyocytes isolated from basal and hypertrophic hearts (Panther database). **(B)** Scheme illustrating formation of gene-related groups for single-cell interactome analysis. **(C)** Enriched gene ontology terms of top differentially genes in Cluster 1 and Cluster 2 cardiomyocytes isolated from hypertrophic hearts (PANTHER and Reactome database). **(D)** Single-cell violin plots illustrate expression of cell-cycle genes in WT and TAC cardiomyocytes. "Normalized counts" refers to sequence counts after size-factor normalization.

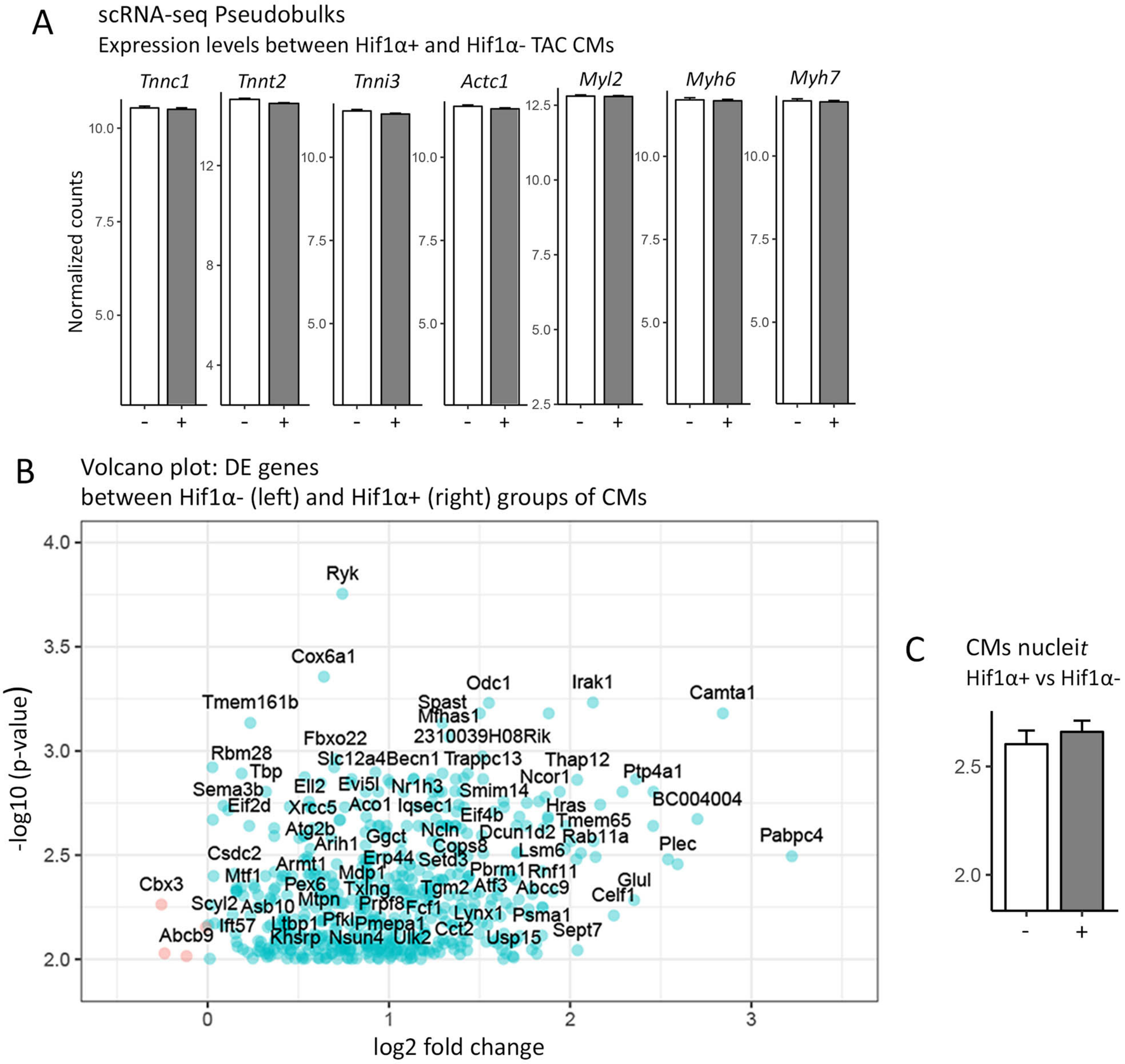

Supplementary Figure 4. Heterogeneity of cardiomyocytes in hypertrophic hearts is driven by hypoxic responses. (A) Volcano plot showing genes differentially expressed in Hif1α^{+} and Hif1α^{-} cardiomyocytes. **(B)** Pseudobulk bar plots demonstrate similar expression of cardiac marker genes in Hif1α^{+} and Hif1α^{-} cardiomyocytes. "Normalized counts" refers to sequence counts after size-factor normalization. **(C)** Bar plots displaying average numbers of nuclei in Hif1α^{+} and Hif1α^{-} cardiomyocytes.

Supplementary data sets (provided as separate excel files)

Supplementary data set 1: Differentially expressed genes between base line and TAC cardiomyocytes.

Supplementary data set 2: Differentially expressed genes between Cluster 1 and Cluster 2 cardiomyocytes.

Supplementary data set 3: Expression of cardiac marker genes between Hif1a+ and Hif1a- cardiomyocytes.

Supplementary data set 4: Differentially expressed genes between Hif1α^+ and Hif1α^- cardiomyocytes.
